# Starting at Birth: An Integrative, State-of-the-Science Framework for Optimizing Infant Neuromotor Health

**DOI:** 10.3389/fped.2021.787196

**Published:** 2022-01-24

**Authors:** Colleen Peyton, Theresa Sukal Moulton, Allison J. Carroll, Erica Anderson, Alexandra Brozek, Matthew M. Davis, Jessica Horowitz, Arun Jayaraman, Megan O'Brien, Cheryl Patrick, Nicole Pouppirt, Juan Villamar, Shuai Xu, Richard L. Lieber, Lauren S. Wakschlag, Sheila Krogh-Jespersen

**Affiliations:** ^1^Department of Physical Therapy and Human Movement Sciences, Northwestern University Feinberg School of Medicine, Chicago, IL, United States; ^2^Institute for Innovations in Developmental Sciences, Northwestern University, Chicago, IL, United States; ^3^Department of Pediatrics, Northwestern University Feinberg School of Medicine, Chicago, IL, United States; ^4^Department of Psychiatry and Behavioral Sciences, Northwestern University Feinberg School of Medicine, Chicago, IL, United States; ^5^Department of Medical Social Sciences, Northwestern University Feinberg School of Medicine, Chicago, IL, United States; ^6^Ann and Robert H. Lurie Children's Hospital, Stanley Manne Children's Research Institute, Chicago, IL, United States; ^7^Shirley Ryan AbilityLab, Chicago, IL, United States; ^8^Division of Rehabilitative Services, Ann & Robert H. Lurie Children's Hospital of Chicago, Chicago, IL, United States; ^9^Department of Dermatology, Northwestern University Feinberg School of Medicine, Chicago, IL, United States; ^10^Department of Physiology, Northwestern University Feinberg School of Medicine, Chicago, IL, United States; ^11^Department of Physical Medicine and Rehabilitation, Northwestern University Feinberg School of Medicine, Chicago, IL, United States

**Keywords:** neuromotor health, infants, physical therapy, transdiagnostic, early intervention

## Abstract

Numerous conditions and circumstances place infants at risk for poor neuromotor health, yet many are unable to receive treatment until a definitive diagnosis is made, sometimes several years later. In this integrative perspective, we describe an extensive team science effort to develop a transdiagnostic approach to neuromotor health interventions designed to leverage the heightened neuroplasticity of the first year of life. We undertook the following processes: (1) conducted a review of the literature to extract common principles and strategies underlying effective neuromotor health interventions; (2) hosted a series of expert scientific exchange panels to discuss common principles, as well as practical considerations and/or lessons learned from application in the field; and (3) gathered feedback and input from diverse stakeholders including infant caregivers and healthcare providers. The resultant framework was a pragmatic, evidence-based, transdiagnostic approach to optimize neuromotor health for high-risk infants based on four principles: (a) active learning, (b) environmental enrichment, (c) caregiver engagement, and (d) strength-based approaches. In this perspective paper, we delineate these principles and their potential applications. Innovations include: engagement of multiple caregivers as critical drivers of the intervention; promoting neuromotor health in the vulnerability phase, rather than waiting to treat neuromotor disease; integrating best practices from adjacent fields; and employing a strengths-based approach. This framework holds promise for implementation as it is scalable, pragmatic, and holistically addresses both the needs of the infant and their family.

## Introduction

Neuromotor health is a critical substrate of infants' development and learning. Early development is a period of great change, vulnerability, and opportunity, making early identification and amelioration of neuromotor risk of the utmost importance. More than 17% of children will have a diagnosed developmental disability (1, 2), many of whom will also have motor challenges. Myriad environmental, genetic, and medical circumstances influence infants' motor development across multiple domains. Given the interdependent nature of these developing abilities, a multi-modal, holistic approach is needed beginning before formal diagnosis.

Early intervention is effective at preventing or mitigating pediatric neuromotor conditions (3). However, many established rehabilitation interventions are limited in that: (1) early transdiagnostic (i.e., those that target multiple conditions, multiple risk mechanisms or vulnerability to risk) approaches lack consensus guidelines (4–6) and (2) they are not tailored to the infant's and/or family's ecology (i.e., unique contexts, values, and needs), contributing to disparities in service access, engagement, and neuromotor outcomes. These limitations have impeded many interventions from actualizing their promise to improve neuromotor outcomes for all infants.

Recent strides have been made in early intervention evidence for infants with motor challenges, including the importance of education and support of caregivers (7, 8), understanding family ecology when setting therapeutic goals (9), infant-initiated movements (9–12), and supporting the transition from hospital to home (9, 13). Building on this foundation, our goal was to reach further into adjacent disciplines of developmental psychology, infant mental health and prevention, and implementation science (14–17) to create a neuromotor intervention framework that incorporates considerations of holistic development, family engagement, implementation, and scalability.

This perspective paper synthesizes diverse literatures, expert panels, and stakeholder feedback as the foundation for a novel framework: the Caregiver engagement, Active leaRning, Environmental enrichment, and Strengths-based framework (CARES). CARES promotes optimized infant neuromotor health, relational health, and family wellbeing during the first year of life. In keeping with the *Healthier, Earlier* vision we have previously articulated (18), the CARES framework promotes beginning intervention at the earliest stage of the risk sequence before conditions are typically diagnosed, promoting neuromotor health via early detection or attenuation of neuromotor delays.

## Methods

### Process to Identify and Refine Common Intervention Principles

Our goal was to synthesize commonalities in: (1) principles, defined as broadly applicable theoretical concepts that form the foundation of interventions and (2) discrete clinical strategies (i.e., actions, skills, or methods utilized by the therapist and caregivers) that underlie successful intervention in infants at increased risk of poor neuromotor health. To extract these principles and strategies, we used the sequenced approach of conducting a literature review, convening expert panels, and encouraging stakeholder engagement. (For detailed methods on how this process was conducted, see Supplementary Material A).

#### Literature Review

Our literature review initially focused on studies examining developmental populations with neuromotor conditions (e.g., cerebral palsy, born preterm, born full-term with brain injury) from traditional fields of rehabilitation science and motor learning. Due to the aforementioned gaps in this literature, we then felt it was critical to include evidence from adjacent fields of developmental psychology, clinical psychology, infant mental health, and prevention-implementation science. This review identified common strategies, constraints and future directions.

#### Expert Panels

We invited distinguished researchers and clinicians to discuss “lessons learned” from their early intervention studies and experiences with our group. We convened 12 scientific exchange panels, organized thematically (Supplementary Material B). The panels comprised 25 individuals from 18 institutions in four countries. Panelists included researchers, physical therapists, physicians, psychologists, and nurses with research and clinical expertise in the domains of neuromotor development, parenting and family-based interventions, implementation science, infant mental health, and neonatal care. The cross-fertilization from these panels provided invaluable insights including pragmatic, real-world considerations that were fundamental to formulation of CARES common principles and strategies. Importantly, intervention scientists provided insight regarding pragmatic approaches and scalability for transdiagnostic interventions (19, 20).

#### Stakeholder Engagement

##### Caregivers

We recruited caregivers with diverse caregiving roles and socioeconomic backgrounds to participate in a panel through Northwestern University's Center for Community Health. Panelists were caregivers of young children with neuromotor risk or diagnosis who shared their perspectives about neuromotor development and intervention services to inform approaches to physical therapy with infants at-risk. Caregivers highlighted the following themes as critical for consideration: (1) Desire for more information and early education about neuromotor development; (2) Value of strengths-based positive, knowledgeable, and effective therapists; and (3) Hope and optimism for future developments in research and interventions to promote neuromotor health.

##### Healthcare Providers

Neonatologists and nursing staff provided perspectives on medical and therapeutic care of high-risk infants. Infant mental health specialists discussed early caregiver-infant relationships and caregiver well-being as factors in infant developmental outcomes and caregiver adherence to intervention efforts. Finally, physical therapists identified potential barriers and facilitators of integrating new principles into practice.

## Results

### Intervention Principles Underlying CARES: An Integrative Framework for Optimizing Infant Neuromotor Health

**Figure 1:**
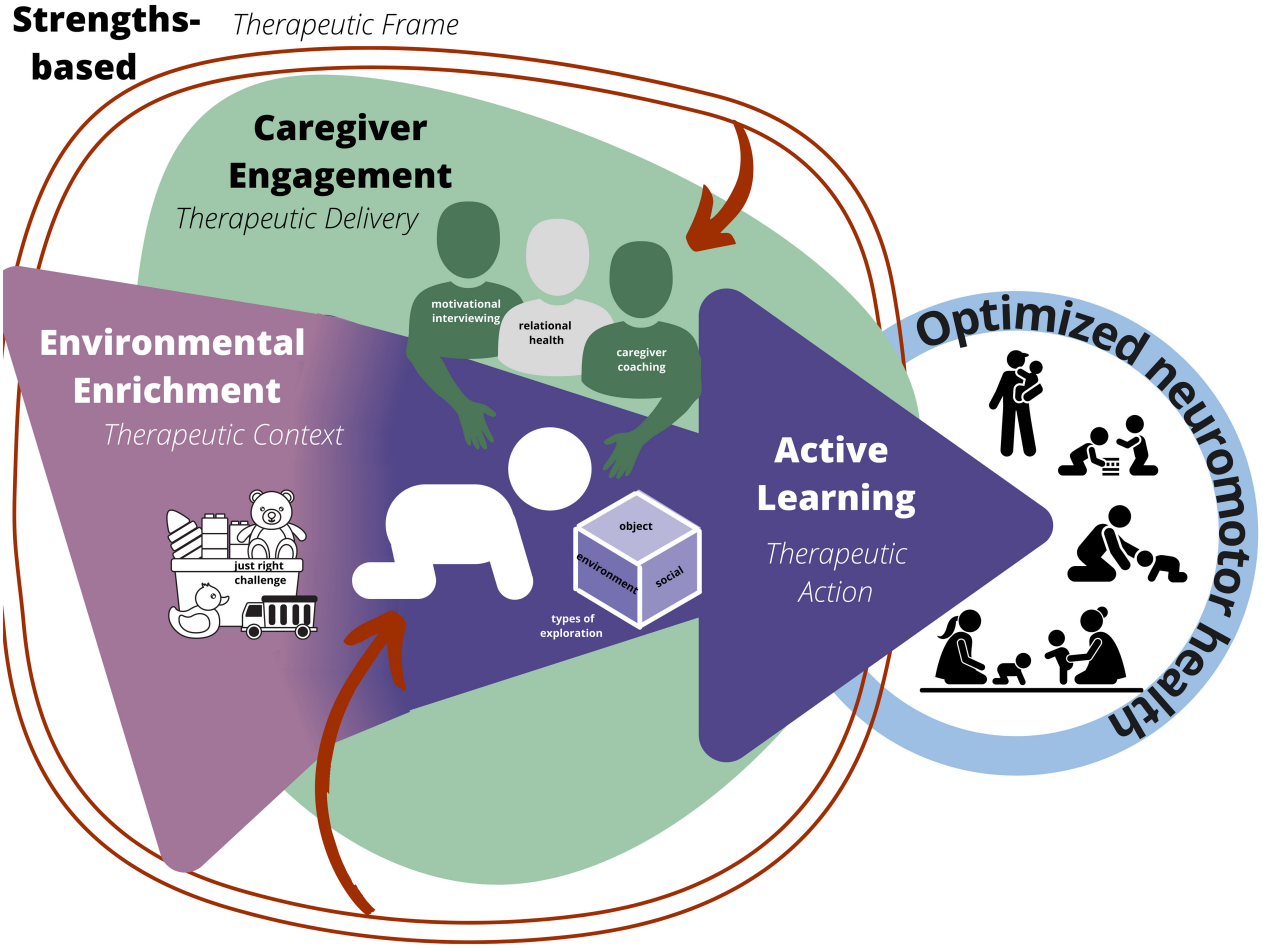
CARES Framework. Active learning is the therapeutic action or mechanism (dark purple), environmental enrichment is the therapeutic context, creating opportunities for action to occur (light purple), caregiver engagement is therapeutic delivery of intervention (green), and strength-based approach is the therapeutic frame (red) influencing each level.

As shown in Figure 1, the CARES principles are connected through a lens of therapeutic action (active learning; mechanism that drives change), therapeutic context (environmental enrichment; environment in which therapy is delivered), therapeutic delivery (caregiver engagement; the way in which the therapeutic dose is provided), and therapeutic frame (strengths-based; the construct that informs treatment). Each of the principles and their respective strategies are defined and discussed in detail in the following sections and outlined in Table 1.

**Table 1:** Heuristic comparison of CARES framework compared to commonly established therapies.

**Principle**	**Therapeutic feature**	**Established therapy**	**CARES framework (definition)**	**Strategies**
Active learning	Therapeutic action	Therapist-led movement treatment	Movement is infant-directed and focuses on exploration of their surroundings	• Wait-Model-Support-Stop Scaffolding • Social, environmental, object exploration
Environment enrichment	Therapeutic context	Therapy clinic or use of equipment and/or toys not accessible for families.	Settings and/or activities are relevant to everyday life Balance task difficulty with achievability through supports, object manipulation, and/or social scaffolding for the infant	• Just-right challenge • Build active learning opportunities into baby's environment • Selection of objects, settings, and caregiver interactions within the infant's family context
Caregiver engagement	Therapeutic delivery	Therapist as expert and caregiver as recipient Focus is child-centered Therapist trains mostly one or “primary” caregiver	Caregivers take an active, leading role in the intervention for their infant Focus on relational health between caregivers and infant Multiple caregivers involved in intervention delivery	• Caregiver coaching (collaborative goal setting, problem-solving, circle of care) • Motivational interviewing • Attentiveness to relational health
Strengths-based framing	Therapeutic frame	Problem-focused Medical, deficit model	Opportunity-focused A balanced approach to bring about change by framing goals and mechanisms using positive attributes and strengths of the infant, caregiver, and family	• Reframing and positive self-talk • Labeled praise and celebration of developmental goals and activities

### Cares Principle 1: Active Learning (Therapeutic Action)

Active learning is defined as infant-directed actions, occurring when an infant moves to explore their environment, including the people and objects around them. Infant motor development results from maturing physiologic systems that are shaped by task-specific experiences and environmental demands (21). Learning and neuroplasticity are maximized when an activity is salient (22), the movement is initiated by the infant (23–25), and the task is repeated with variable strategies and errors (21). This current view contrasts directly with established approaches in physical therapy intervention in which therapist-handling of the infant aims to inhibit movement patterns deemed “abnormal” and/or to facilitate movement patterns defined as “normal,” creating a passive partnership in which the infant is the recipient of a therapist-led handling treatment (26). Passive activities may engage aspects of the sensory system (27), but are less likely to create new motor connections in the infant's brain. Passive activities are also less likely to result in the cognitive and social growth associated with infant-driven learning (28–30).

The adoption of current neuroscience-oriented motor learning theory in physical therapy has led to newer approaches that promote infant active exploration. This active learning is critical in early development for establishing motor, cognitive, and social competences (29–32).

To maximize an infant's active learning, the infant must be given time to act. If this is not successful or the infant does not make any attempts, a therapist may model the behavior for them to see before offering help to complete a task (i.e., “Wait-Model-Support-Stop scaffolding”). Modeling actions relies on cognitive mechanisms, such as imitation (33) that support the process of active learning. Finally, learning is limited in stressful situations (34, 35), so if an infant becomes upset the activities should be stopped or modified.

Active learning can be observed in three types of exploration: (1) object exploration (e.g., toys, food, utensils), (2) social exploration (e.g., caregivers, siblings), and (3) environmental exploration (e.g., sand, grass, slippery surface). The specific type of exploration can be used as a strategy to incorporate treatment into daily routines, centered around caregiver goals and priorities.

### Cares Principle 2: Environmental Enrichment (Therapeutic Context)

Environmental enrichment is defined in the CARES framework as the creation of a space or activity within the infant's natural environment, which is designed to pair the level of difficulty of a task with the infant's ability to complete the task. Socio-cultural influences, and variations in the physical environment influence and shape motor responses and learning in human infants (36). Targeted environmental enrichment strategies can improve motor outcomes in infants with neuromotor risk (37). Everything around an infant that they may see, hear, or interact with defines their environment. As such, caregivers and therapists alike may not be aware that their presence impacts the environment of the infant as well as their actions, and the design of intervention.

The key strategy of environmental enrichment is targeted selection of objects or toys, settings, body positions, and caregiver interactions that facilitate achieving the infant's and/or family's goals. The selection of objects and settings can enhance active learning if they are easily accessible within the infant's everyday routine, generating numerous opportunities to explore. In contrast, other models of therapy that include specific therapeutic equipment available only in a specialized therapeutic care setting may narrow the opportunity for repetition.

The specific choice of everyday objects can be used to meet family goals. For instance, if caregivers would like the infant to be able to reach with both hands, the environment can be enriched by considering the properties of the toys in the infant's world; a toy that is larger or more fluid is more likely to be played with using both hands (38). Objects or supports in the environment can also help an infant gain access to a skill that would otherwise be too difficult. For instance, the use of a reclined seat may provide an infant with the opportunity to reach for and manipulate an object by reducing the postural demands on an infant who is not yet able to independently support themselves in an upright position (39). Another strategy that can be used to enrich an infant's environment is to provide a “just-right challenge” (40), tailoring the level of difficulty of a task to the infant's ability level to create a targeted context for an infant to explore and learn. When an infant engages in a just-right challenge, they are often active and playful, resulting in higher amounts of problem-solving, repetition, and practice. By enriching the infant's natural environment, new opportunities for learning and problem-solving are afforded to the infant in a therapeutic context and the broader family ecology.

### Cares Principle 3: Caregiver Engagement (Therapeutic Delivery)

Caregiver engagement is defined as a collaborative coaching model in which therapists and caregivers are equal partners, and families are fully engaged as decision-makers and participants in the infant's intervention. Whereas many therapy models (41) view the therapist as the “expert” and caregivers as “recipients” of the intervention, this caregiver-led approach builds on families' capacity to successfully and confidently implement the intervention and enhance their infants' development. Caregiver engagement is a collaborative process whereby the family and therapist work together to set goals for therapeutic activities based on the caregivers' priorities for their infant and family (42), the unique resources available to and challenges faced by the family, and the therapist's experience and knowledge. Capacity-building also means welcoming the infant's entire “circle of care” into the intervention—that is, engaging as many caregivers as the family deems appropriate. This reduces burden on the primary caregiver [often the mother (43–45)] by distributing responsibilities and, importantly, increasing dosage. Caregiver engagement includes the following strategies: caregiver coaching, motivational interviewing, and attentiveness to relational health.

*Caregiver coaching* centers the caregiver as the interventionist and promotes capacity-building by encompassing collaborative goal-setting with and coaching of the caregiver to implement the intervention. Caregiver-led and caregiver-implemented approaches are effective in early intervention studies, and collaborative coaching between caregivers and therapists increases caregivers' confidence and competence in intervention planning and implementation (46, 47).

*Motivational interviewing* is a widely validated therapeutic skill used to elicit caregivers' unique motivations, treating them as the experts of their own experience, and working *with* them instead of *on* them (48, 49). Motivational interviewing involves non-judgmental collaboration, techniques to elicit caregivers' ideas and solutions, and respect of caregivers' autonomy for decision-making (48, 49). Many studies show that motivational interviewing increases treatment engagement and retention, which in turn leads to better health outcomes (50–52). Therapists use this skill to identify potential challenges to engagement by approaching caregivers with the assumption that intervention will be most effective when it is tailored to the demands and environments of each family, and when priorities identified by caregivers receive central focus. As such, caregivers feel understood and build greater rapport with the therapist. The therapeutic delivery is one of empathy and reflection, exploring what works and what does not, and supporting caregivers' autonomy and self-determination. Fundamentally, this approach helps caregivers identify their own capacity to engage in the intervention activities with their infant.

*Relational health* refers to the quality of the infant's earliest relationships and is foundational for optimal growth and development (53–56). The relational health emphasis in early intervention recognizes that (a) infants thrive most within secure, responsive relationships and (b) caregivers are best equipped to be agents of the intervention in the context of responsive, collaborative relationships with the therapist. Relationship focused intervention increases caregiver engagement and satisfaction, decreases therapist burnout, improves caregiver-infant interactions, and ultimately improves developmental outcome (14). The shift from child-centric to relationship-focused intervention requires therapist competence in not only neuromotor therapy, but also in intervention practices that support the caregiver-infant relationship, as the foundation of developmental growth. Key therapeutic actions include: promoting caregiver feelings of confidence and competence, fostering collaborative caregiver-therapist relationships via empathic listening and reflection, and having the capability to sensitively manage emotionally fraught interactions or engagement challenges in a manner that promotes trust and engagement.

Taken together, caregiver engagement strategies holistically promote family active participation in the intervention, accounting for each family's unique ecological context, and supporting delivery of the intervention to the infant.

### Cares Principle 4: Strengths-Based Approach (Therapeutic Frame)

A strengths-based approach emphasizes the positive attributes, capacities, and resources of the infant and their family. This approach contrasts with a typical medical model that is pathology-driven, focused on correcting deficits (57–59). Importantly, a strengths-based approach does not minimize neuromotor risk or delay, but rather views them as multifaceted, comprising strengths, supports, and challenges. Clear communication about areas of growth is paramount and framed in a positive, capacity-building manner. This paradigm shift is evident in early childhood education and social services (60, 61), and may increase support and participation amongst families and their children with complex needs, including those at risk for poor neuromotor health (62).

Specific strategies used to operationalize the strengths-based principle include techniques that enhance the strengths of families, including cultural strengths. One example is the use of labeled praise to encourage an infant's behavior or skill and/or empower caregiver confidence. Labeled praise provides a clear connection to a behavior or action of the infant to the desired outcome (63). This technique can also be used to increase caregiver confidence and engagement by recognizing their efforts and affirming their capacity in caring for their infant (64).

Positive reframing broadens caregivers' perspective of themselves, their circumstances, or their infant, allowing family members to see beyond the challenges or perceived deficits (65). Reframing interventions using a strengths-based approach places the focus on opportunities for growth, rather than problems to be fixed (60). A traditional problem-based approach can contribute to a power imbalance between the family and therapist when the therapist imposes their own ideas to solve problems instead of enabling families to develop their knowledge and skills as an opportunity for learning in an ecologically relevant way (62).

Employing a strength-based approach as the therapeutic frame of the intervention also invites caregivers to think more optimistically about their infant's potential and imbues a sense of strength and competency in their care of their infant and in celebrating infant gains.

## Discussion

We followed a comprehensive, integrative, transdisciplinary approach to identify common principles and strategies underlying effective intervention components. We drew from the fields of developmental psychology, infant mental health and prevention- implementation science to develop a framework that is transdiagnostic, sensitive to diverse families' needs, and scalable.

CARES principles are interrelated, and implementation of each principle facilitates the others. Active learning is at the center of the intervention as the driver of therapeutic change, a concept that is now emerging in the field. Employing strategies of environmental enrichment for the infant and caregivers creates more opportunities for active learning in naturally occurring contexts. Engaging caregivers and building their capacity to participate in their respective caregiving roles will enhance their confidence and competence to effectively deliver the intervention within their unique family ecology. Finally, the strengths-based approach pervades each level of the therapeutic action, environment, and delivery as the therapist works with families to recognize their strengths, including their support systems and available resources to best support their caregiving capacity.

The use of caregiver engagement as an approach to therapeutic delivery is not often described in the fields of rehabilitation science or motor learning. Because of the age-specific context of infant intervention, the relationship between the infant and caregivers and ecology of the family system must be considered for successful and scalable intervention delivery. By harnessing methods from the fields of infant mental health and developmental psychology, the CARES framework focuses on a relational health model, rather than an infant-centered model, further enhancing the environment in which the infant and family co-develop. The therapist plays a role in understanding and supporting these principles, creating a positive and supportive therapeutic alliance with the family, so that new opportunities for learning and problem-solving are afforded to both the infant and caregivers and can be tailored toward family-specific goals.

This paper lays out the CARES framework as an integrative novel conceptual approach. We believe this is a promising avenue to explore but note that our model development process was comprehensive, though not exhaustive. The inclusion of local clinical stakeholder feedback in the tailoring of the model is a strength. Still to enhance generalizability of the model, integration of feedback from a broader representation of national stakeholders is needed. Finally, we have not yet tested the CARES framework and thus it is still theoretical.

The next critical step will for application of the CARES framework will be rigorous scientific testing for addressing neuromotor risk from an early age using a variety of study designs including randomized control trials, implementation frameworks, and comparative effectiveness paradigms. We found extensive value in the contributions from stakeholders with a wide range of lived experiences, and would encourage the use of community participatory research design to ensure an equitable approach that can be implemented in diverse situations. Finally, although this study was targeted toward children at high risk for delay based on preterm birth or early adverse events, these principles are likely to extend to other infants based on a broad range of perinatal risk as well as those with conditions diagnosed at birth.

## Data Availability Statement

The original contributions presented in the study are included in the article/Supplementary Material, further inquiries can be directed to the corresponding author.

## Author Contributions

CP, TS, AC, SK-J, LW, AB, EA, JH, and JV contributed to conception and design of this perspective. CP, TS, and AC wrote the first draft of the manuscript. All authors contributed to manuscript revision, read, and approved the submitted version.

## Funding

This work was generously supported by the Patrick G and Shirley W Ryan Foundation. CP receives support from National Center for Advancing Translational Sciences, Grant KL2TR001424. SX recognizes support from the Hartwell Foundation.

## Conflict of Interest

The authors declare that the research was conducted in the absence of any commercial or financial relationships that could be construed as a potential conflict of interest.

## Publisher's Note

All claims expressed in this article are solely those of the authors and do not necessarily represent those of their affiliated organizations, or those of the publisher, the editors and the reviewers. Any product that may be evaluated in this article, or claim that may be made by its manufacturer, is not guaranteed or endorsed by the publisher.
