# Supplementary material for: Starting at Birth: An Integrative, State-of-the-Science Framework for Optimizing Infant Neuromotor Health
Source: Front Pediatr. 2022 Jan 24;9:787196. doi: 10.3389/fped.2021.787196 (PMC8820372; doi:10.3389/fped.2021.787196)
Supplement: Supplementary file 1 [file Data_Sheet_1.docx]

**Supplemental Material:**

**Supplemental Material A: Scientific Exchange Panel Series**

Procedure prior to each panel: We provided a template to all panelists asking them to address the overview of their intervention; core components, mechanisms, and outcomes measured; adaptations needed to start earlier, whether it could apply transdiagnostically and involve parent-delivery; tailoring for individual patients/families; barriers to implementation, and unpublished “lessons learned.”

**Listed below are dates, speakers, topics and suggested pre-readings to audience members**

**Scientific Exchange Panel Series Kick-Off**

*July 21^st^, 2020*

- Introduction and discussion with key stakeholders
- Structure and Scientific Exchange Panel project implementation

**Exploratory Play & Parent Interaction to Promote Early Neuromotor Adaptation**

*July 28^th^, 2020*

- Regina Harbourne, PT, PhD, PCS, FAPTA, Assistant Professor at Duquesne University
- Ann-Christin Eliasson, PhD, Senior Professor at the Karolinska Institute in Sweden
- Barbara Sargent, PhD, PT, PCS, Assistant Professor at the University of Southern California
- Pre-readings:
  - Sitting Together and Reaching to Play (START-Play): Protocol for a Multisite Randomized Controlled Efficacy Trial on Intervention for Infants with Neuromotor Disorders
  - The effectiveness of Baby-CIMT in infants younger than 12 months with clinical signs of unilateral-cerebral palsy; an explorative study with randomized design
  - Efficacy of baby-CIMT: study protocol for a randomized controlled trial on infants below age 12 months, with clinical signs of unilateral CP
  - In-Home Kicking-Activated Mobile Task to Motivate Selective Motor Control of Infants at High Risk of Cerebral Palsy: A Feasibility Study

**Early mobility measurement and learning**

*August 4^th^, 2020*

- **Diane Damiano, PhD, PT, FAPTA Senior Investigator at the National Institutes of Health**
- **Jill Heathcock, MPT, PhD, Associate Professor at The Ohio State University**
- **Linda Fetters, PhD, PT, FAPTA Professor at the University of Southern California**
- Pre-readings
  - Daily and weekly rehabilitation delivery for young children with gross motor delay: a randomized clinical trial protocol (The DRIVE study)
  - Early, Accurate Diagnosis and Early Intervention in Cerebral Palsy: Advances in Diagnosis and Treatment
  - Proposing “E” Words to Summarize Key Intervention Principles for Infants at Risk for Cerebral Palsy: Systematic Review of Systematic Reviews (under review)

**Supporting parents and infants in the NICU and transition to home**

August 17^th^, 2020

- **Alicia Spittle, PhD, Professor at the Murdoch Children’s Research Institute, Melbourne, Australia**
- **Stacey Dusing, PT, PhD, Associate Professor and Director of Pediatric Research at the University of Southern California**
- Pre-readings
  - The role of social risk in an early preventative care programme for infants born very preterm: a randomized controlled trial
  - Efficacy of Supporting Play Exploration and Early Development Intervention in the First Months of Life for Infants Born Very Preterm: 3-Arm Randomized Clinical Trial Protocol
  - Knowledge Translation Lecture: Providing Best Practice in Neonatal Intensive Care and Follow-up: A Clinician-Researcher Collaboration

**State of the science: Early intervention in infants at high risk of neuromotor differences**

*August 24^th^, 2020*

- **Alicia Spittle, PhD, Professor at the Murdoch Children’s Research Institute, Melbourne, Australia**
- **Cathy Morgan, PhD; B.App.Sc., Senior Research Fellow at the Cerebral Palsy Association, Sydney, Australia**
- Pre-readings
  - Early developmental intervention programmes provided post hospital discharge to prevent motor and cognitive impairment in preterm infants
  - Effectiveness of motor interventions in infants with cerebral palsy: a systematic review

**Implementation science: enabling clinicians to utilize the best available evidence in real-world practice**

*September 14^th^, 2020*

- **Suzanne Davis Bombria, PT, c/NDT**
- **Ginny Paleg, PT, DScPT**
- **Nathalie Maître, MD, PhD, Director of the NICU Follow-up Program, Medical Director of NICU Developmental Therapies, and Assistant Professor at Nationwide Children’s Hospital**
- **Suggested Reading**
  - The Impact of Neuro-Developmental Treatment on the Performance of Daily Living Tasks by Children with Cerebral Palsy - Pilot Studies in Measuring NDT Outcomes
  - State of the Evidence Traffic Lights 2019: Systematic Review of Interventions for Preventing and Treating Children with Cerebral Palsy
  - Early childhood constraint therapy for sensory/motor impairment in cerebral palsy: a randomised clinical trial protocol

**From Bench to Bedside to Policy: Translational Approaches to Early Intervention**

*September 21^st^, 2020*

- **Cindy Miles, PT, PhD, PCS, CNDT, pediatric private practice**
- **Sue Greaves, B.App. Sc.(OT), MOT, PhD, Manager of Occupational Therapy at The Royal Children’s Hospital, Melbourne, Australia**
- Gay Girolami, PT, PhD, Clinical Professor and Director of Professional Studies at the University of Illinois at Chicago
- Pre-readings
  - A Systematic Review of Part C Early Identification Studies
  - **REACH: study protocol of a randomized trial of rehabilitation very early in congenital hemiplegia**
  - Efficacy of a Neuro-Developmental Treatment Program to Improve Motor Control in Infants Born Prematurely
  - Study protocol: an early intervention program to improve motor outcome in preterm infants: a randomized controlled trial and qualitative study of physiotherapy performance and parental experiences
  - Early Parent-Administered Physical Therapy for Preterm Infants: A Randomized Controlled Trial
  - Effects of a Parent-Administered Exercise Program in the Neonatal Intensive Care Unit: Dose Does Matter- A Randomized Controlled Trial

**NICU nurse engagement in interventions for infant neuromotor health**

*September 29^th^, 2020*

- **Rosemary White-Traut, PhD, RN, FAAN, Director of Nursing Research at Children’s Wisconsin, Professor Emerita at the University of Illinois at Chicago, Adjunct Professor at the University of Washington, Seattle, Rush University, and the Medical College of Wisconsin**
- **Amy Manion PhD, APRN, CPNP-BC, Associate Professor at Rush University**
- **Sue Horner, MS, APRN-NP,CNS, RNC-NIC, Lurie Children’s hospital**
- **Pre-reading**
  - **Impact of an integrated mother-preterm infant intervention on birth hospitalization charges**
  - Barriers to and Intervention that Increase Nurses’ and Parents’ Compliance with Safe Sleep Recommendations for Preterm Infants
  - Setting the Stage for Successful Oral Feeding: The Impact of Implementing the SOFFI Feeding Program with Medically Fragile NICU Infants

**Overview of key implementation designs and their application to the Corbett Ryan Project**

*October 5^th^, 2020*

- J.D. Smith, PhD, Associate Professor at the University of Utah
- **Pre-reading**
  - **The Implementation Research Logic Model: a method for planning, executing, reporting, and synthesizing implementation projects**

**Strategies for engaging diverse families and caregivers in prevention**

*October 13^th^, 2020*

- **Melanie Pellecchia, PhD, BCBA, NCSP, Assistant Professor at the University of Pennsylvania Perelman School of Medicine**
- **Cady Berkel, PhD, Associate Professor at the Arizona State University**
- J.D. Smith, PhD, Associate Professor at the University of Utah
- **Pre- reading**
  - **Mind the gap: an intervention to support caregivers with a new autism spectrum disorder diagnosis is feasible and acceptable**
  - Enrollment and Attendance in a Parent Training Prevention Program for Conduct Problems
  - An individually tailored family-centered intervention for pediatric obesity in primary care: study protocol of a randomized type II hybrid effectiveness-implementation trial (Raising Healthy Children study)
  - Motivation Interviewing and Caregiver Engagement in the Family Check-Up 4 Health

**Varied approaches in diverse populations, settings, and resources**

*October 19^th^, 2020*

- Kath Benfer, PhD, Post-Doctoral Research Fellow at the Queensland Cerebral Palsy and Rehabilitation Research Centre
- **Cathy Morgan, PhD; B.App.Sc., Senior Research Fellow at the Cerebral Palsy Association, Sydney, Australia**
- Laura Prosser, PT, PhD, Assistant Professor at the Perelman School of Medicine at the University of Pennsylvania and The Children's Hospital of Philadelphia
- Jana Iverson, PhD, Professor at the University of Pittsburgh
- **Suggested Reading**
  - **Community-based parent-delivered early detection and intervention programme for infants at high risk of cerebral palsy in a low-resource country (Learning through Everyday Activities with Parents (LEAP-CP): protocol for a randomized controlled trial**
  - GAME (Goals-Activity-Motor Enrichment): protocol of a single blind randomized controlled trial of motor training, parent education and environment enrichment for infants at high risk of cerebral palsy
  - Feasibility and preliminary effectiveness of a novel mobility training intervention in infants and toddlers with cerebral palsy
  - iMOVE: Intensive Mobility training with Variability and Error compared to conventional rehabilitation for young children with cerebral palsy: the protocol for a single blind randomized controlled trial
  - Early Motor and Communicative Development in Infants with an Older Sibling with Autism Spectrum Disorder

**NICU2Home: A novel app for mothers and fathers engagement in the NICU transition**

*October 27^th^, 2020*

- Craig Garfield, MD, Lurie Children’s Hospital, Professor at the Northwestern University Feinberg School of Medicine
- Pre-reading
  - **Supporting parents of premature infants transitioning from the NICU to home: A pilot randomized control trial of a smartphone application**
  - Father-Inclusive Perinatal Parent Education Programs: A Systematic Review

**Auditory-Tactile-Visual-Vestibular (ATVV) Intervention**

*December 14, 2020*

- **Rosemary White-Traut, PhD, RN, FAAN, Director of Nursing Research at Children’s Wisconsin, Professor Emerita at the University of Illinois at Chicago, Adjunct Professor at the University of Washington, Seattle, Rush University, and the Medical College of Wisconsin**
- Pre-reading

**Mental health training for developmental allied healthcare professionals**

*January 14, 2021*

- Orna Zohar, Efrat Sher- Censor, Judith Elata, University of Haifa, Israel
- Suggested Reading
  - A mental health course for developmental allied healthcare professionals: an exploration of potential effectiveness

**SUPPLEMENTAL MATERIAL B**

**THERAPIST TRAINING:**

The training was a total of ~21 hours and included asynchronous and synchronous content. See below training schedule for time and topics covered. Participants generally preferred synchronous discussion and role-playing (versus asynchronous activities) for learning. Block training listed below refers to age-specific phases of the intervention.

**Intervention Training Schedule:**

Day 1:

| Time | Format | Topic |
| --- | --- | --- |
| 9-9:50am | Synchronous (Zoom) | Introductions, background of study |
| 10-11am | Synchronous (Zoom) | Orientation to manual; orientation to Canvas |
| 11am-12pm | *Asynchronous (Canvas)* | Introduction   Course introduction videos   Innovations of Project Corbett intervention |
| 12-1pm: Lunch |  |  |
| 1pm-2pm | *Asynchronous (Canvas)* | Scientific Rationale for Block Structure   Rationale for blocks video    Timeline for Intervention and Assessments   Intervention and assessment timeline   Goal Attainment Scale |
| 2pm-3pm | Synchronous (Zoom) | Q&A, Canvas issues |

Day 2

| Time | Format | Topic |
| --- | --- | --- |
| Evening (anytime!)  2 hours total | *Asynchronous content (Canvas)* | Administering a Protocolized Intervention   Delivery of a protocolized Intervention   Clinical reasoning flow for each session   REDCap orientation    Common Elements of the Intervention   Caregivers as active participants in NICU care   Positive framing and strengths based model   Active exploration    Block A Specific Training   Block A specific training |

Day 3:

| Time | Format | Topic |
| --- | --- | --- |
| 9am-11:00am (with 10min break) | Synchronous (Zoom) | Block A: recognizing signals, talking with families, delivering standardized care, goal setting, etc. (role play and discussion) |
| 11am-12pm | Synchronous (Zoom) | Using REDCap forms and NICU activities |
| 12pm-1pm Lunch |  |  |
| 1pm-3pm | Synchronous (Zoom) | Relational Health and Motivational Interviewing Part 1 |
| 3pm-4pm | Synchronous (Zoom)    *Optional asynchronous content located under “Resources” Module* | Intervention forms: Recommended Activities, Caregiver check-in, GAS goals, Therapist daily documentation and reflection, Home environment checklist, Technology checklist, PRIME |

Day 4:

| Time | Format | Topic |
| --- | --- | --- |
| Evening (anytime!)  2 hours total | *Asynchronous content (Canvas)* | Block B Training   Block B specific training    Block C Training   Block C specific training    Standard Operating Procedures   Block A study procedures   Block B-D study procedures   Equipment setup    Fidelity   Fidelity overview |

Day 5:

| Time | Format | Topic |
| --- | --- | --- |
| 9am-9:50 | Synchronous (Zoom) | Block B and Block C specific training, talking with families, delivering standardized care, goal setting, etc (role play and discussion) |
| 10am-10:50 | Synchronous (Zoom) | Fidelity: what, why, and how |
| 11am-12pm | Synchronous (Zoom) | Discussion, Q&A, weekly schedule once pilot begins |
| 12pm-1pm Lunch |  |  |
| 1pm-3pm | Synchronous (Zoom) | Motivational interviewing and relational health: part 2 |
| 3pm-4pm | Synchronous (Zoom) | Delivering a protocolized intervention check-in    Training wrap-up and Q&A |
